# CpG Demethylation Enhances Alpha-Synuclein Expression and Affects the Pathogenesis of Parkinson's Disease

**DOI:** 10.1371/journal.pone.0015522

**Published:** 2010-11-24

**Authors:** Lumine Matsumoto, Hiroshi Takuma, Akira Tamaoka, Hiroshi Kurisaki, Hidetoshi Date, Shoji Tsuji, Atsushi Iwata

**Affiliations:** 1 Division of Neuroscience, Department of Neurology, Graduate School of Medicine, The University of Tokyo, Bunkyo, Tokyo, Japan; 2 Department of Molecular Neuroscience on Neurodegeneration, Graduate School of Medicine, The University of Tokyo, Bunkyo, Tokyo, Japan; 3 Department of Neurology, University of Tsukuba, Tsukuba, Ibaraki, Japan; 4 Department of Neurology, National Hospital Organization Tokyo Hospital, Kiyose, Tokyo, Japan; 5 PRESTO, Japan Science and Technology Agency (JST), Kawaguchi, Saitama, Japan; Brigham and Women's Hospital, Harvard Medical School, United States of America

## Abstract

**Background:**

Alpha-synuclein (*SNCA*) gene expression is an important factor in the pathogenesis of Parkinson's disease (PD). Gene multiplication can cause inherited PD, and promoter polymorphisms that increase *SNCA* expression are associated with sporadic PD. CpG methylation in the promoter region may also influence *SNCA* expression.

**Methodology/Principal Findings:**

By using cultured cells, we identified a region of the *SNCA* CpG island in which the methylation status altered along with increased *SNCA* expression. Postmortem brain analysis revealed regional non-specific methylation differences in this CpG region in the anterior cingulate and putamen among controls and PD; however, in the substantia nigra of PD, methylation was significantly decreased.

**Conclusions/Significance:**

This CpG region may function as an intronic regulatory element for SNCA gene. Our findings suggest that a novel epigenetic regulatory mechanism controlling *SNCA* expression influences PD pathogenesis.

## Introduction

Parkinson's disease (PD) (MIM168600) is a neurodegenerative disease characterized by akinesia, rigidity, tremor, and postural instability. The prevalence is more than 1% of the population over 65 years old [1]. Most of the PD cases are sporadic, although 10% is hereditary and caused by mutations in *SNCA*, *PARK2*, *LRRK2*, *PINK1*, *UCHL1*, or *DJ1*, in the majority of cases [2]. Mutations in these genes are occasionally found in sporadic PD; however, the majority of sporadic cases show no evidence of mutation in any of the known causative genes.

The pathological hallmark of PD is Lewy bodies (LB), which are intracellular inclusion bodies consisting of aggregated alpha-synuclein. Abnormal accumulation of aggregated protein is closely associated with the pathogenesis of many neurodegenerative diseases. The precise mechanism of aggregation remains unknown, but increased expression of aggregation-prone proteins can lead to their aggregation. For example, in Down syndrome, duplication of the 21^st^ chromosome, which contains the amyloid beta precursor protein (APP) gene, leads to accumulation of amyloid beta and Alzheimer's disease pathology [3]. In rare cases of PD, duplication or triplication of *SNCA* gene leads to alpha-synuclein accumulation, with triplication producing a more severe phenotype than duplication, suggesting that *SNCA* expression level determines the severity of the pathology [4], [5], [6]. Animal models of neurodegenerative disorders are generated by over-expression of causal genes, further supporting the conclusion that increased gene expression is related to pathogenesis [7]. Additional evidence indicates that *SNCA* promoter polymorphisms increases alpha-synuclein expression and increases susceptibility to sporadic PD [8], [9], [10]. On the basis of these reports, we hypothesized that there may be another unknown mechanism for increased *SNCA* expression in PD.

In addition to promoter polymorphisms, epigenetic modification can alter downstream gene expression. Epigenetic regulation includes histone modification and DNA methylation, of which CpG island methylation can be gene-specific; in several different cancers, CpG methylation inhibits binding of the transcription machinery, causing silencing of a specific oncogene, which leads to carcinogenesis [11], [12], [13]. In central nervous system disorders, CpG methylation has been associated with psychiatric disorders, such as autism and schizophrenia [14].

We sought to identify CpG islands in the *SNCA* gene, wherein methylation status was associated with alpha-synuclein expression. For this purpose, we had to find cell lines which express endogenous alpha-synuclein at sufficient levels to support a comparative analysis. To our surprise, we confirmed that non-neuronal 293 cells express alpha-synuclein protein, and expressed dopamine receptor genes DRD1 and DRD2. *SNCA* gene expression is affected by cell stress, such as serum deprivation, Interleukin-1β [15], Lipopolysaccharide (LPS) [16], nerve growth factor (NGF) [17], basic fibroblast growth factor (bFGF) [18], 1-methyl-4-phenylpyridine (MPP+) [19], and dopamine [20]. By using these potential alpha-synuclein up-regulators, we have identified an *SNCA* CpG island that controls *in vitro* alpha-synuclein expression, and then analyzed postmortem brain tissues to find PD-specific *SNCA* promoter demethylation in the substantia nigra.

## Methods

### Cell culture

HeLa and 293 (HEK-293) cells purchased from ATCC (Manassas, VA, USA) were grown in Dulbecco's modified eagle medium (Sigma, St. Louis, MO, USA) with 10% fetal bovine serum (BIO Whittaker, Walkersville, MD, USA). SH-SY5Y cells also purchased from ATCC were grown in Dulbecco's modified Eagle's medium nutrient mixture F-12 HAM (Sigma), with 0.15% sodium bicarbonate solution (Sigma) and 1 mM sodium pyruvate (Sigma), at 37°C in 5% CO_2_ in air. Plasmid transfection was performed using Lipofectamine 2000 (Invitrogen, Carlsbad, CA, USA), according to the manufacturer's protocol. Interleukin-1β, Lipopolysaccharide (LPS) (L2654, L6529), human basic fibroblast growth factor (bFGF), human nerve growth factor (NGF)-β, dopamine hydrochloride, and haloperidol were purchased from Sigma. Cell viability assays (MTS (3-(4,5-dimethylthiazol-2-yl)-5-(3-carboxymethoxyphenyl)-2-(4-sulfophenyl)-2H-tetrazolium) assays) were performed with CellTiter 96 AQ_ueous_ One Solution Reagent (Promega, Madison, WI, USA), according to the manufacturer's protocol. OD at 490 nm was measured on a plate reader Spectramax plus 384 (Molecular Devices, Sunnyvale, CA, USA).

### Protein extraction

Cells were lysed in 50 mM Tris-HCl 7.5, 150 mM NaCl, 1% NP40, 0.5% sodium deoxycholate, and 1 mM ethylenediaminetetraacetic acid (EDTA) supplemented with protease inhibitor cocktail (complete Mini, EDTA-free, Roche Diagnostics, Mannheim, Germany). After SDS-PAGE, proteins were transferred to an Immobilon transfer membrane (Millipore, Bedford, MA, USA). For visualization, horseradish peroxidase-conjugated secondary antibodies were used with ECL Western Blotting Detection Reagents (GE Healthcare UK limited, Buckinghamshire, UK), and detected with LAS-3000 mini (Fuji film, Tokyo, Japan).

### Nucleic acid analysis

DNA was extracted using a DNeasy Blood & Tissue kit (QIAGEN, Hilden, Germany). RNA was extracted by TRIZOL® reagent (Invitrogen, Carlsbad, CA, USA). Reverse transcription was performed with ReverTraAce (Toyobo, Osaka, Japan) following manufacturer's protocol. Probes for quantitative RT-PCR are as follows, (*SNCA* forward primer: 5′-CCAGTTGGGCAAGAATGAAGAA-3′, reverse primer: 5′-CTTGATACCCTTCCTCAGAAGGC-3′, TaqMan probe: 5′-[FAM]AATTCTGGAAGATATGCCTGTGGATCCTGA[TAMRA]-3′; DRD1: Hs01024463_m1, DRD2: Hs00377719_g1, DRD3: Hs00945865_mH, ACTB: 4326315E (for reference)) (Applied Biosystems, Carlsbad, CA, USA). Analysis was performed on an ABI 7900HT instrument (Applied Biosystems). Relative expression levels were calculated using comparative Ct method.

### Methylation analysis

#### Bisulfite sequencing

Methprimer (http://www.urogene.org/methprimer/index1.html) was used to design primers for bisulfite sequencing (5′-GGGAGGTTAAGTTAATAGGTGGTAA-3′ and 5′-CCCTCAACTATCTACCCTAAACAAAC-3′).

Bisulfite conversion was performed with the EpiTect Bisulfite kit following the manufacturer's “low-concentration” protocol (QIAGEN). For each samples, 100ng of genomic DNA was used and incubated at 95°C for 5min, 60°C for 25min, 95°C for 5min, 60°C for 85min, 95°C for 5min and 60C°C for 175min. Converted product was purified following the instructions of the Epitect Bisulfite kit. One µL from the final 20 µL elute was PCR-amplified with LA Taq (Takara) following the manufacturer's mixture condition and 30 PCR cycles of 98°C 10sec, 55.5°C (for CpG-1) or 63°C (for CpG-2) 30sec, 68°C 60sec. The PCR product was cloned to pCR′-TOPO vector (Invitrogen). At least 20 clones from each experiment were sequenced by M13 reverse or M13 forward (−20) primers. There are 21 CpH sequences in the region. We used sequence results up to 3 unconverted CpHs for further analysis. For each samples, patterns of methylation and unconverted CpHs were used to detect similar clones. Up to four overlapping clones were allowed for measuring CpG methylation. Data analysis and methylation patter figures were generated using QUMA software [21].

#### Methylated-CpG Island Recovery Assay

A MethylCollector kit (Active Motif, Carlsbad, CA, USA) was used to perform the methylated-CpG island recovery assay (MIRA). The experiment was performed as instructed by the manufacturer. The methylated DNA target fragment was detected with PCR primers 5′-AGCTGCGTTTGGCAAATAAT-3′ and 5′-GCCCAAGTGTGATGGTTCTAA-3′.

### Dual-Luciferase assay

CpG-2 was amplified by primers 5′- TGTGGAGAAGCAGAGGGACT -3′, 5′- ACAGGTTGATGGTGGAAAG -3′ and cloned into pMOD shLuc (InvivoGen, San Diego, CA, USA) which encode firefly luciferase and the entire plasmid was demethylated in JM110 (Stratagene, La Jolla, CA, USA). *In vitro* CpG methylation was performed by incubation with M.SssI (New England Biotechnology, Cambridge, MA, USA) for 1 h at 37°C. Methylated and non-methylated plasmids were transfected into 293 cells with pGL4.74 [hRluc/TK] (Promega) to use sea pansy luciferase as an internal control calibrator. Dual-luciferase activity was measured using the Pick&gene luciferase detection kit (Toyo B-Net co, Tokyo, Japan) on a plate reader Phelios AB-2350 (ATTO, Tokyo Japan). Firefly activity was normalized by sea pansy activity to calibrate background and transfection efficiency.

### Postmortem brain

Postmortem brains were obtained at University of Tokyo Hospital, University of Tsukuba Hospital, and National Hospital Organization Tokyo Hospital, with written consent from patients' families and approval of the ethical committees of each facility. Specimens were maintained at −80°C until analysis. Pathological diagnosis was made by neuropathologists using hematoxylin-eosin, Nissl, silver staining together with immunostaining by phosphorylated tau (AT8, Innogenetics) and phosphorylated alpha-synuclein antibodies (psyn#64, Wako, Japan) following the established criterias [22], [23].

### Statistic analysis

Statistical analysis was performed with the aid of Instat3 software (Graphpad Software, Inc, LaJolla, CA). Significance was tested with ANOVA and Student's t-test.

## Results

### Dopamine alters *SNCA* CpG island methylation and enhances alpha-synuclein expression

Our first goal was to identify the region of the *SNCA* CpG island responsible for transcription regulation. We tested various human cell lines for endogenous alpha-synuclein expression. Immunoblotting revealed that 293 cells, although not neuronal, expressed a significant amount of alpha-synuclein. In fact, the expression in 293 cells was considerably greater than in SH-SY5Y cells, which are often considered to be dopaminergic; thus, we decided to use 293 cells for further analysis.

**Figure 1:**
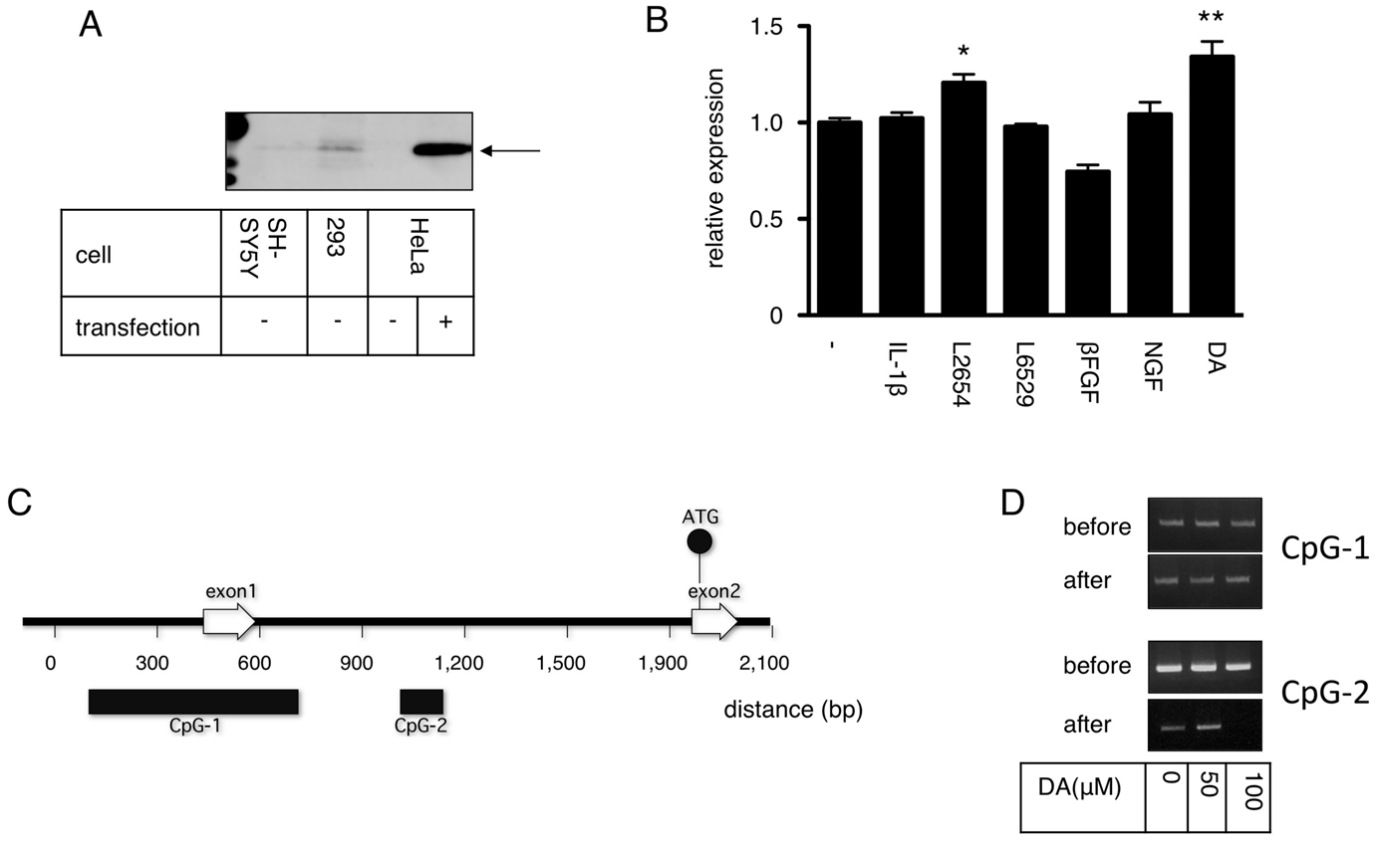
Identification of SNCA CpG islands. **A**: 293 cells express endogenous alpha-synuclein Immunoblot of cell lysates from SH-SY5Y, 293, and HeLa cells with or without over-expression of wild-type alpha-synuclein (transfection − and +). Arrow indicates alpha-synuclein. **B**: Quantification of alpha-synuclein expression in the presence of various stimuli. DMSO (−), IL-1β (100 ng/mL), lipopolysaccharides (L2654 and L6529 1 µg/mL), βFGF (100 ng/mL), NGF (100 ng/mL) and dopamine (DA 50 µM) were added to 293 cells for 48 h and relative expression of alpha-synuclein was compared by quantitative RT-PCR. Relative amounts of alpha-synuclein mRNA were normalized to DMSO = 1.0, * P<0.01, ** P<0.001. **C**: Structure of the SNCA CpG island Entrez gene NC_000004.11 ID was analyzed by CpG island search software (http://www.uscnorris.com/cpgislands2/cpg.aspx.). Non-coding exon1 and coding exon2 are indicated by open arrows. The translation start ATG codon (methionine) located at exon2 is indicated by a closed circle. Two separate CpG islands are indicated by closed bars. **D**: Methylated-CpG island recovery assay of dopamine treated cells. 293 cells treated with or without dopamine were lysed and methylated. CpGs were enriched by MBD2 protein. Methylated CpG-1 or CpG-2 was detected by PCR before and after enrichment.

Using 293 cells, we tested various drugs that have been reported to alter alpha-synuclein expression. Of 6 reagents tested, 1 lipopolysaccharide and dopamine caused a significant increase in the production of alpha-synuclein (Figure 1B). Because dopamine seemed to enhance alpha-synuclein expression more than the lipopolysaccharide, we chose to analyze its effect on *SNCA* CpG island methylation.

The *SNCA* gene has two CpG islands, predicted by CpG detection software (Figure 1C). One of the CpG islands (CpG-1) is located at the first exon, which is not a coding region for alpha-synuclein. The second CpG island (CpG-2) is located in the first intron. We analyzed the methylation status of CpG-1 and -2 by MIRA to test the effect of dopamine. CpG-1 methylation remained unchanged whereas CpG-2 methylation was decreased by the addition of 100 mM dopamine (Figure 1D). Since MIRA is a robust but not a sensitive method for measuring methylation, we next evaluated methylation with the more sensitive bisulfite sequencing method.

**Figure 2:**
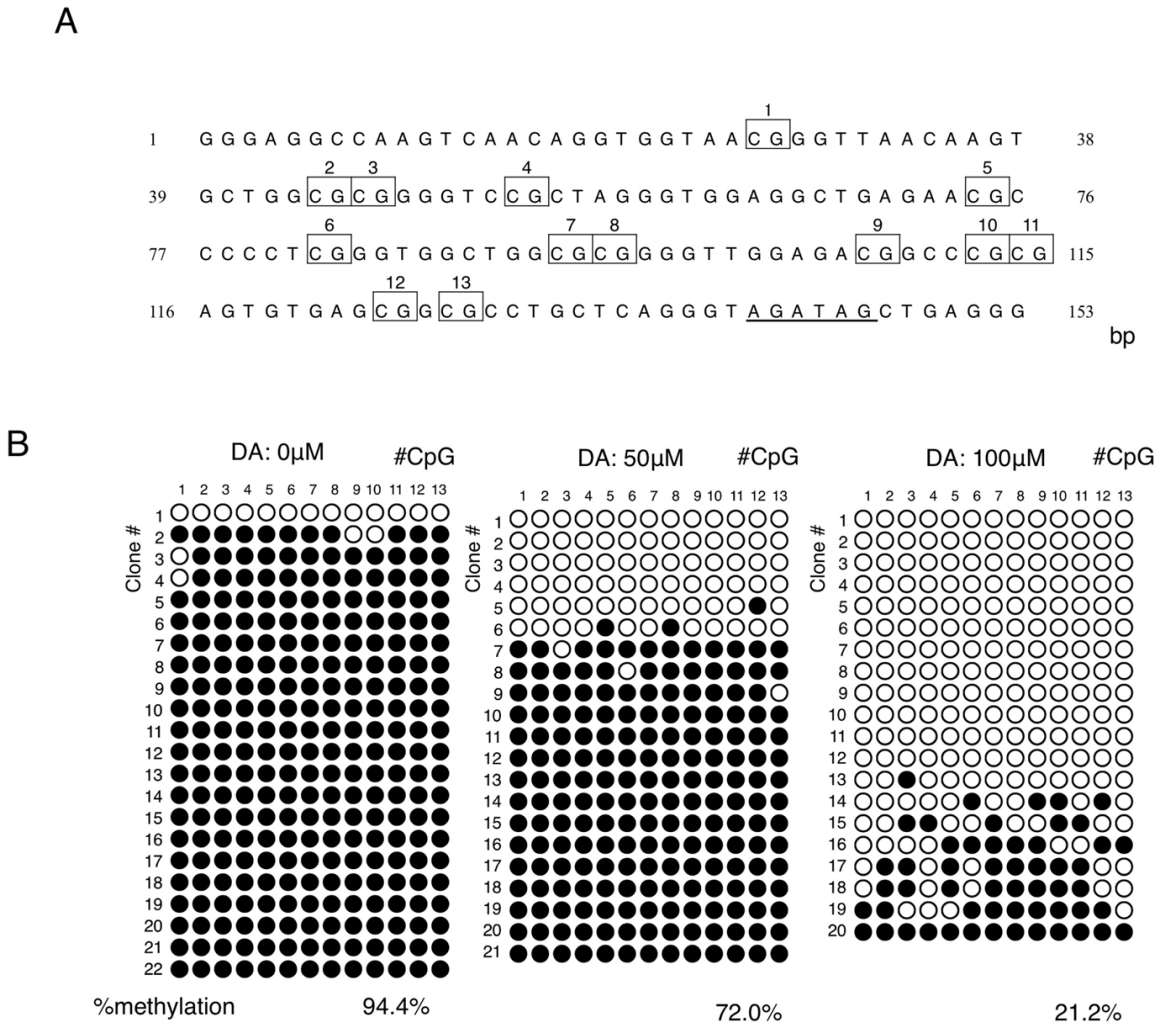
Dopamine induced demethylation of CpG-2. **A**: Location of CpGs in the second SNCA CpG island. There are 13 CpGs in the CpG-2 region. Each CpG is labeled above according to its positions from the 5′ end of the sequence. GATA transcription factor recognition sequence is shown with an under bar. **B**: Bisulfite sequence analysis of the CpG-2 region. Dopamine (DA; 0, 50, and 100 µM) was added to 293 cells. DNA was purified and bisulfite sequence analysis was performed. At least 20 clones from each dopamine-treated group were analyzed. Open circles indicate unmethylated CpGs, whereas closed circles indicate methylated CpGs. The degree of methylation was calculated as methylated CpG/total CpG and shown below (% methylation).

CpG-2 has 13 CpG sequences (Figure 2A). CpG-2 was 94.4% methylated at baseline, and decreased in a dose-dependent manner with the addition of dopamine (0 mM, 94.4%; 50 mM 72.0%; 100 mM 21.2%) (Figure 2B). Bisulfite sequencing analysis of CpG-1 revealed no significant change in methylation with the addition of dopamine (0 mM, 7.2%; 50 mM 3.3%; 100 mM 6.7%, figure not shown).

**Figure 3:**
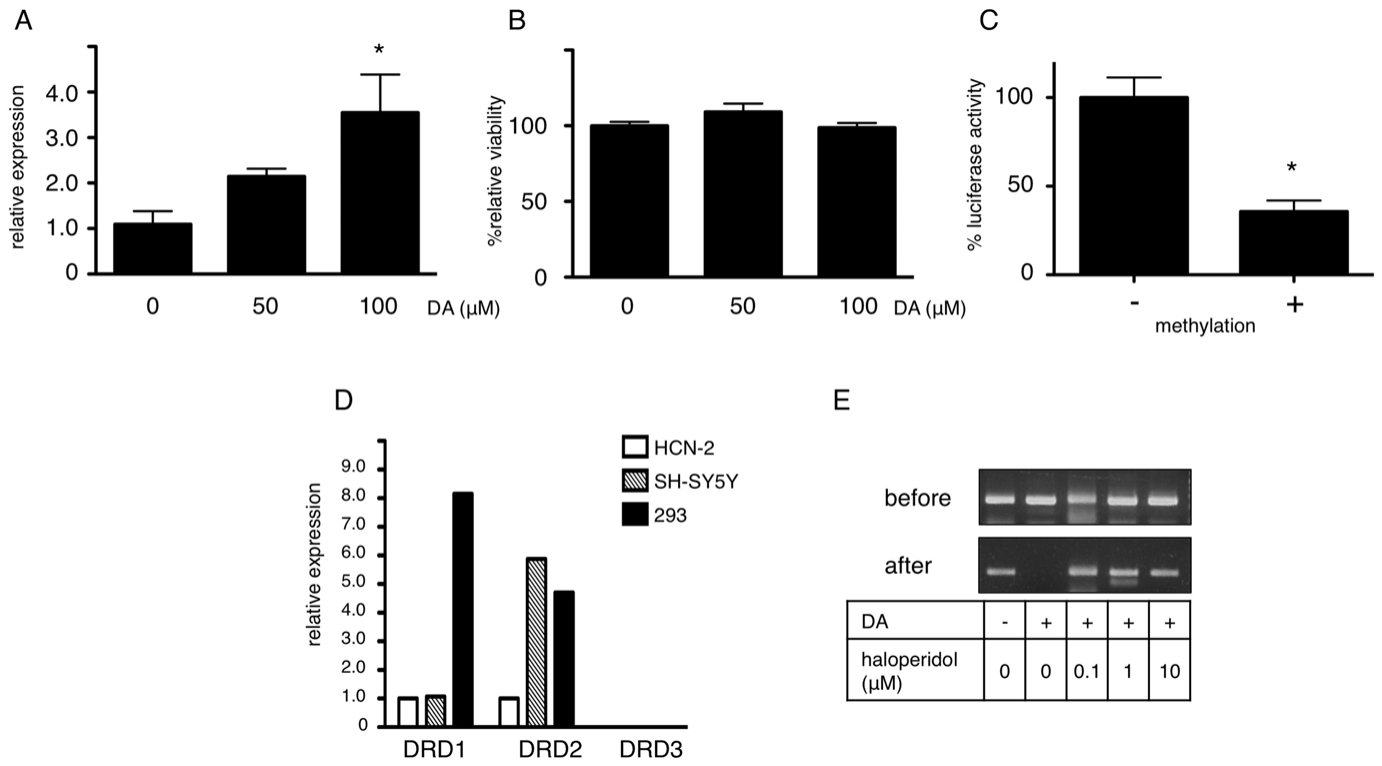
CpG-2 demethylation up-regulates SNCA expression through dopamine receptor signaling. **A**: Dose dependent up-regulation of alpha-synuclein by dopamine. RNA was extracted from cells treated with the indicated dopamine concentration for 48 h and quantitative PCR was performed. The relative amount of alpha-synuclein mRNA against beta-actin was normalized to dopamine = 0. * P<0.05 vs. DA = 0. **B**: Dopamine does not affect cell viability. MTS assay was performed after addition of dopamine for 48 h. P = 0.1559 with ANOVA. **C**: Methylation of CpG-2 suppresses downstream gene expression. Luciferase assays were performed after transfection of a CpG-free firefly luciferase vector harboring the CpG-2 region at the 5′ of the luciferase gene. The vector was treated with (+) or without (-) CpG methyltransferase prior to transfection. Sea pansy luciferase was co-transfected as an internal control. Relative luciferase activity was calculated by dividing firefly luciferase activity by sea pansy luciferase activity and normalized to methylation (−) as 100%. Bars indicate standard error. * P = 0.0025. **D**: 293 cells express D1 and D2 dopamine receptors. Expression of DRD1, −2 and −3 were analyzed in HCN-2, SH-SY5Y, and 293 cells by quantitative RT-PCR. Relative amounts of each receptor are normalized to HCN-2 = 1. **E**: A D2 receptor antagonist ameliorates the demethylation effect of dopamine. Dopamine (DA)- and haloperidol-treated 293 cells were lysed and methylated CpGs were enriched by MBD2 protein. Methylated CpG-2 was detected by PCR before and after enrichment.

We next tested the correlation between dopamine induction of alpha-synuclein expression and CpG-2 demethylation. Addition of 100 mM dopamine further increased the expression of alpha-synuclein mRNA (Figure 3A). Since alpha-synuclein expression is affected by the cellular stress response [24], we tested whether dopamine caused cellular stress at the experimental concentration, and found that 100 mM dopamine did not alter cellular viability, as measured by the MTS method (Figure 3B). We conclude, therefore, that demethylation was not caused non-specifically by dopamine toxicity, but is the result of a more direct mechanism. Finally, we tested whether CpG-2 methylation was essential for downstream gene expression. *In vitro* methylated CpG-2 had a significant negative effect on down-stream gene expression, as measured by luciferase assays (Figure 3C). Thus, we concluded that CpG-2 methylation was essential for alpha-synuclein expression.

### Methylation status of CpG-2 controls alpha-synuclein expression through dopamine receptor signaling

We next tried to understand the underlying mechanism of CpG-2 demethylation. Since 293 cells are not neuronal and there is little knowledge regarding their response to dopamine, we tested 293 cells for expression of dopamine receptors. Quantitative PCR clearly showed that 293 cells expressed DRD1 (D1 receptor) and DRD2 (D2 receptor), at levels comparable to those of dopaminergic SH-SY5Y cells (Figure 3E). To determine whether demethylation of *SNCA* CpG-2 was receptor mediated, we added haloperidol, a D2 receptor antagonist, along with dopamine, and found that haloperidol clearly inhibited the effect of dopamine (Figure 3F). These results strongly suggested that the effect of dopamine on CpG-2 demethylation was a D2 receptor-mediated phenomenon, rather than a direct effect of dopamine on the genome.

### Significant demethylation of CpG-2 in the substantia nigra of PD patients

Since the CpG-2 region was associated with increased expression of alpha-synuclein *in vitro*, we next analyzed the methylation level of this region in the postmortem brain. Twelve brains from PD, Dementia with Lewy Bodies (DLB), and 7 from non-neurodegenerative patients were analyzed. The summary of clinical information and the availability of the brain regions from each case are shown in Tables 1 and 2.

**Table 1:** Summary of the clinical information from the postmortem brains.

Case No.	age at death	sex	diagnosis	disease duration (y)	post mortem time (h)	brain weight (g)	L-DOPA	anterior cingulate				putamen				substantia nigra			
								clones	overlap			clones	overlap			clones	overlap		
									2	3	4		2	3	4		2	3	4
1	69	M	PD	15	18	1390	+	23	3	2	1								
2	71	F	PD	NA	NA	NA	+	21	6	2	0	18	3	1	0	17	0	2	0
3	73	M	PD	5	1.5	NA	+	20	4	1	0	12	1	1	0				
4	76	M	PD	31	2.4	NA	+	18	3	1	0	21	1	1	1				
5	78	M	PD	28	24	NA	+	20	3	0	0	18	2	1	0	14	1	1	0
6	78	M	PD	NA	NA	1100	+	16	0	1	1	17	4	0	0				
7	79	M	PD	30	16	1455	+	23	3	0	2								
8	80	M	PD	NA	6	1510	+	16	2	1	0								
9	81	M	PD	3	1.4	1140	+	21	3	1	0								
10	83	F	PD	4	4	1180	+	22	2	2	1	16	3	1	0				
11	85	M	PD	30	17	1300	+	21	0	1	2					15	2	0	0
12	64	F	DLB	5	5	950	+	21	2	0	2	13	1	1	0	18	0	1	2
13	57	F	lupus encephalitis	NA	3	NA	−	19	3	1	1	20	2	0	0	19	3	2	1
14	59	F	gastric cancer	NA	4	990	−	15	2	0	1	13	3	0	0	21	3	1	1
15	66	F	ovarian cancer	NA	12	NA	−	23	5	1	0	16	3	0	0				
16	70	F	POEMS syndrome	NA	16	NA	−	17	4	0	0	19	2	0	0				
17	76	M	prostate cancer	NA	3	NA	−	22	2	1	1								
18	76	M	esophagus cancer	NA	NA	NA	−	22	2	1	0					17	3	1	0
19	80	M	subdural hematoma	NA	7	985	−	24	2	2	0								
20	87	F	peritonitis	NA	2.5	NA	−	17	4	0	0								

Analyzed regions are shown at the right three columns, where the numbers of the analyzed clones are listed. For each samples, patterns of methylation and unconverted CpHs were used to detect similar clones. Up to four overlapping clones were allowed for measuring CpG methylation. Numbers of the overlapping clones are also shown.

**Table 2:** Statistical representation of the clinical information on the postmortem brains.

	Age at death	Brain weight	Post-mortem time	Disease duration
	(y.o.)	(g)	(h)	(yrs)
Non-PD/DLB control	73.4±9.3	987.5±3.5	7.42±5.48	―
PD/DLB	76.4±6.1	1251.9±191.7	9.53±8.3	16.8±12.8

Age at death (P = 0.41), brain weight (P = 0.1), postmortem time (P = 0.59) to autopsy are not significantly different between disease and control groups.

**Figure 4:**
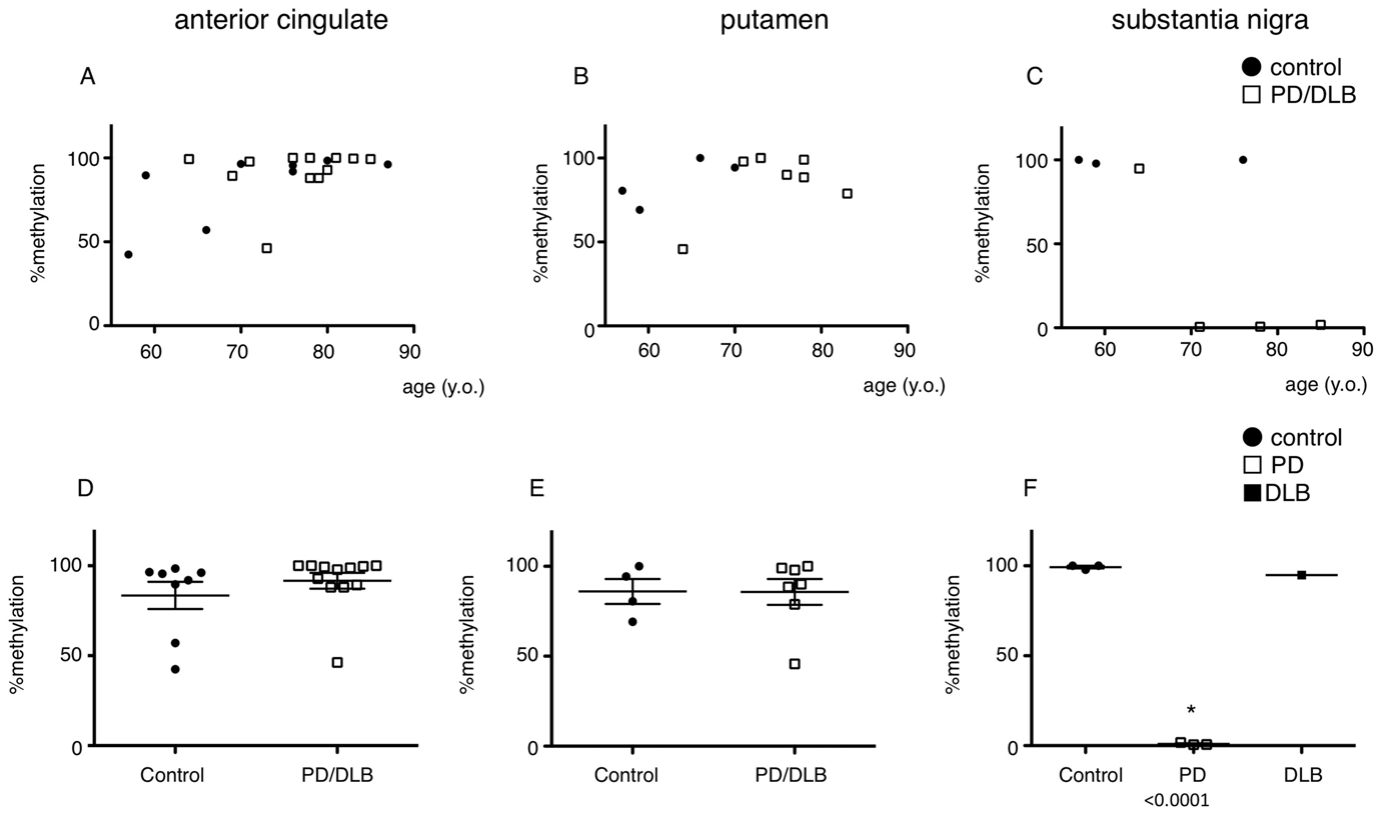
Analysis of CpG-2 methylation level in various brain regions. **A–C**: CpG-2 methylation was analyzed by bisulfite sequencing and plotted against age. Data from anterior cingulate cortex (control: n = 8, PD/DLB: n = 12) (A), putamen (control: n = 4, PD/DLB: n = 7) (B), and substantia nigra (control; n = 3, PD/DLB: n = 4) (C) are shown. Closed circle = controls, open box = PD/DLB. In figure 4C, age and % methylation had no significant correlation P = 0.2321. D–F: CpG-2 methylation was analyzed by bisulfite sequencing method and plotted by disease groups. Data from anterior cingulate cortex (D), putamen (E) and substantia nigra (F) are shown. Closed circle = controls, open box = PD, closed box = DLB P<0.0001 vs. control.

**Figure 5:**
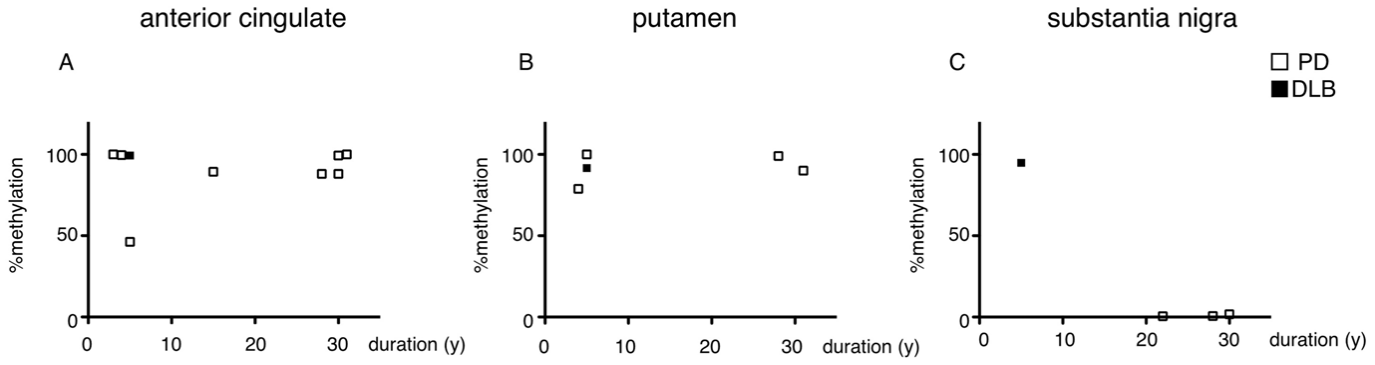
Specific CpG-2 demethylation in substantia nigra of Parkinson's disease patients. CpG-2 methylation in the anterior cingulate cortex (A), putamen (B), and substantia nigra (C) of PD (open boxes) and DLB (closed boxes) are plotted against disease duration.

Bisulfite sequence analysis was performed for all available brain regions and the % methylation was plotted against various clinical parameters (Figures S1, S2, S3). When we compared methylation status of the anterior cingulated cortex and the putamen in controls and PD/DLB patients, there were no significant correlations (Figure 4A, B, D, E). However, methylation status of the substantia nigra was significantly lower in PD patients than in the controls (Figure 4F) and was unaffected by age (Figure 4C). Finally, we plotted % methylation against disease duration and found that in “normally” methylated DLB case, the disease duration was shorter than the PD cases (Figure 5A–C) suggesting extension of Lewy body pathology correlates with CpG-2 hypomethylation.

## Discussion

### Identification of CpG-2 as *SNCA* regulator

We analyzed the effects of several reagents on alpha-synuclein expression in 293 cells and found that dopamine had the most pronounced effect without affecting cell viability. We also tried to screen reagents that altered CpG methylation along with increased alpha-synuclein expression level in SH-SY5Y cells; however, reagents we used did not increase its expression level without affecting cell viability significantly. To avoid the possibility of CpG demethylation by cell death, we decided to use 293 cells instead of SH-SY5Y cells.

Using dopamine as a modulator of *SNCA* expression, we searched for a CpG island which exhibited altered methylation which correlated with changes in gene expression. Of 2 CpG islands identified in the *SNCA* gene, we located the second CpG island at the first intronic region, and found that it showed a significant decrease in methylation, associated with induction of alpha-synuclein expression. This was first identified by MIRA method [25] and was confirmed by bisulfite sequencing [26], which yielded comprehensive results.

CpG-1 lies from the 5′ end of *SNCA* exon1 throughout the exon, and CpG-2 is located at intron1. Methylated CpGs in promoter regions regulate the expression of downstream genes via inhibition of transcription factor binding. Although the precise regulatory mechanism for *SNCA* transcription is unknown, transcription factors GATA at CpG-2 (Figure 2A) [27] and ZSCAN21 [28] at upstream of CpG-2 have been reported to bind at intron1. In addition to this, gamma-synuclein, a close homolog of alpha-synuclein, is reported to have a similar regulatory region [29]; we thus concluded that CpG-2 in intron1 was a methylation dependent regulatory element of *SNCA*. We found that 293 cells expressed D1 and D2 receptors, which supported the rationale for our investigation of dopamine-mediated CpG demethylation in this cell line although they are not neuronal cells. Additional experiments showed that haloperidol, the D2 receptor antagonist, clearly inhibited demethylation by dopamine; thus we concluded that this demethylation was mediated by dopamine receptor D2. D2 receptor activation is coupled with G protein G_αi_, which increases phosphodiesterase activity. CaMK and the protein kinase C pathway are activated by degradation of cAMP by phosphodiesterase [30]. This signaling pathway may be responsible for CpG-2 demethylation.

### CpG-2 is significantly hypomethylated only in the substantia nigra of PD patients

Previous studies have compared alpha-synuclein expression in postmortem brain or peripheral blood from PD patients, with inconsistent results, partly because of artifacts of sample quality and degradation of RNA extracts from postmortem brain tissue [31], [32], [33]. In contrast to these, CpG methylation is reported to be stable up to 48 hours post mortem [34] and we also confirmed that in mice brain, methylation level of SNCA CpG were stable up to 24 hours (Figure S5). These reports support the relevance of investigating *SNCA* CpG methylation in postmortem brains to derive conclusions regarding gene expression. *SNCA* CpG-2 methylation levels differed among subjects and regions tested (Figure 4). This result was not surprising, since a report regarding analysis of CpG methylation in various CpGs suggested their diversity by age, sex, and race [35]. However, the result from substantia nigra was quite striking; the methylation level was almost zero in the PD group, whereas in the control group it was almost 100%, suggesting that methylation in the substantia nigra is an important component of PD pathogenesis. For concerns about DNA quality from substantia, nigra, we tested in several ways to assure that those were comparable between controls and PDs (Figure S4).

The demethylatyion of CpG-2 could be occurring in both neurons and glial cells especially because in the late disease course, massive neuronal loss can occur. However, this does not indicate that our findings is unrelated to the direct pathogenesis, since it has widely recognized that alpha-synuclein aggregation are observed also in the astrocytes and oligodendrocytes of PD brains [36], [37], [38], [39]. In neurodegenerative diseases, it is still a mystery that neurons in similar functions start to degenerate simultaneously regardless of their spatial location in the brain. We can speculate that in the early developmental stage, even before the neuronal and the glial cells are differentiated, epigenetical abnormality can occur. After long incubational time, slightly over-expressed alpha-synuclein can accumulate enough in the neurons to cause PD, while in the glial cells, efficient protein degradation system or the cell division might help them to prevent the accumulation.

Global CpG methylation decreases with age, and specific genes, such as cancer suppressors, are down-regulated by decreased promoter region CpG island methylation [40]. Our findings suggest that in PD patients, normally occurring *SNCA* CpG-2 hypermethylation does not occur, causing over-expression of alpha-synuclein and leading to its accumulation which, in turn, causes PD.

## Supporting Information

Figure S1 **Methylation patterns from anterior cingulate samples generated by QUMA software.** Data are presented similarly as in figure 2B. CpG islands are horizontally and the clones are vertically plotted. × stands for unconverted cytosines.
(TIF)

Figure S2 **Methylation patterns from putamen samples.**
(TIF)

Figure S3 **Methylation patterns from substantia nigra samples.**
(TIF)

Figure S4 **Quality check of genomic DNA from substantia nigra.** **A**: Electrophoresis of 500ng genomic DNA from different substantia nigra. No degradation was observed. **B**: PCR amplification of CpG-2 region from genomic DNA extracted from substantia nigras. Equal amplification of the region was seen with different PCR cycles. Primers used to clone CpG-2 fragment for luciferase assay experiment was used.
(TIF)

Figure S5 **CpG methylation is stable up to 24hours of postmortem time.** **A**: A sequence of mouse SNCA CpG island analyzed is shown. There are 13 CpGs. **B**: C57/BL mice were sacrificed and kept at RT for indicated hours then the SNCA CpG methylation was analyzed by bisulfite sequencing. Primers used to amplify bisufite converted DNA are 5′-ATAAAATTGAGGGTTTTTTGAATT-3′and 5′-CTCTAACTCCCTAACTCCTTCAC -3′. Three mice were used for each time points. Bars are SEs.
(TIF)
