# Supplementary figures and images for: CpG Demethylation Enhances Alpha-Synuclein Expression and Affects the Pathogenesis of Parkinson's Disease
Source: PLoS One. 2010 Nov 24;5(11):e15522. doi: 10.1371/journal.pone.0015522 (PMC2991358; doi:10.1371/journal.pone.0015522)

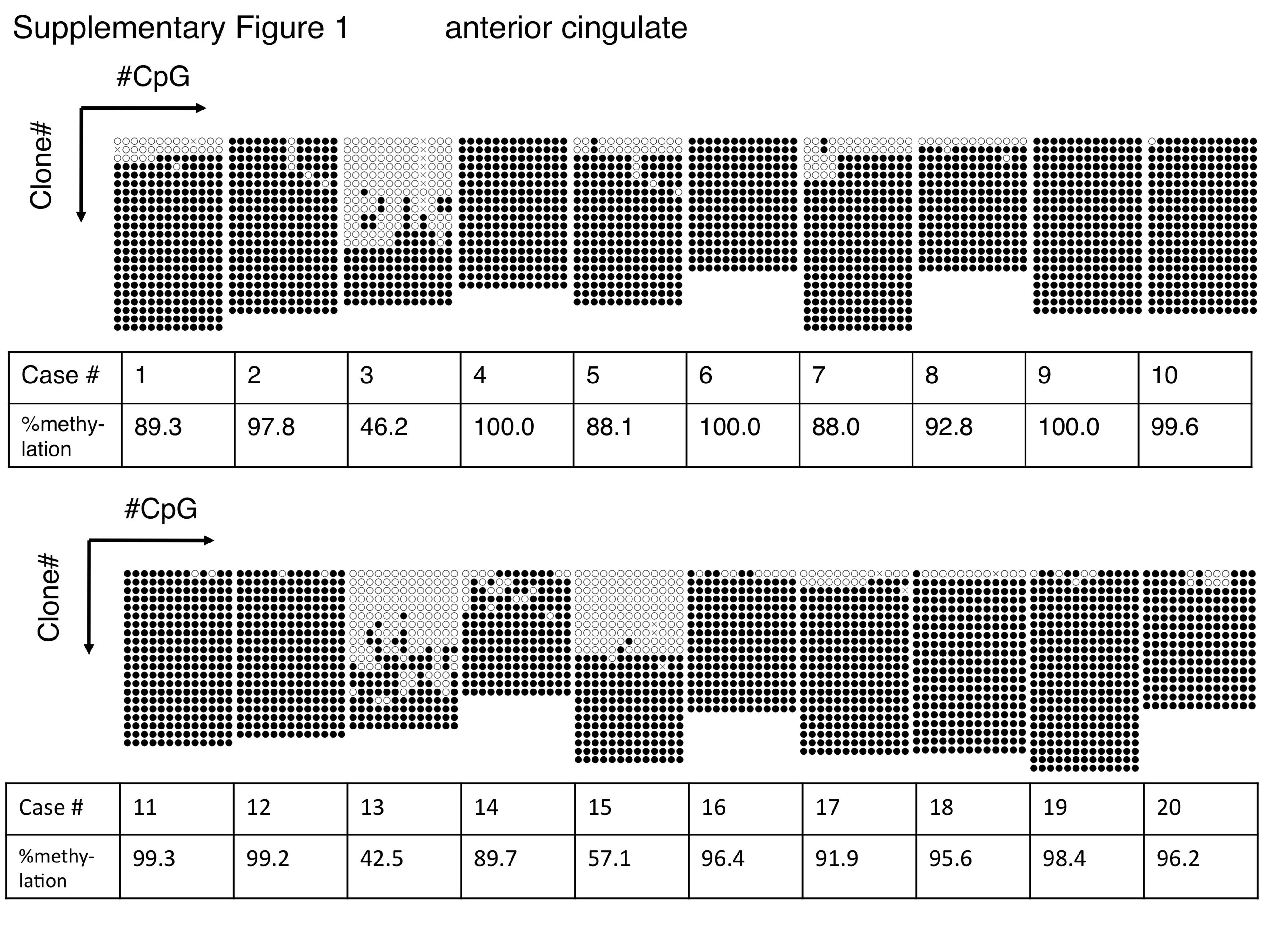

Supplement: Figure S1 — Methylation patterns from anterior cingulate samples generated by QUMA software. Data are presented similarly as in figure 2B. CpG islands are horizontally and the clones are vertically plotted. × stands for unconverted cytosines. (TIF) [file pone.0015522.s001.tif]

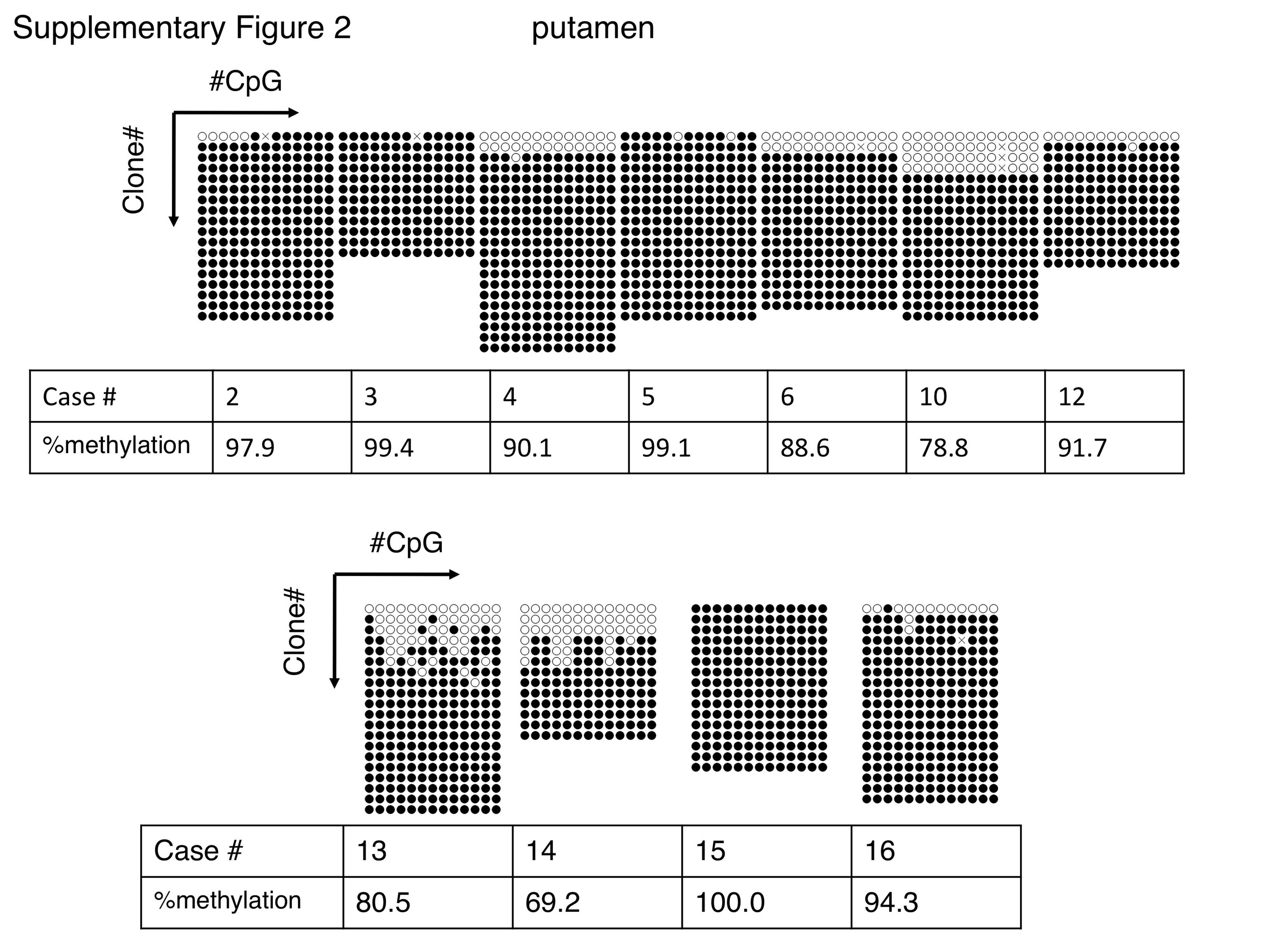

Supplement: Figure S2 — Methylation patterns from putamen samples. (TIF) [file pone.0015522.s002.tif]

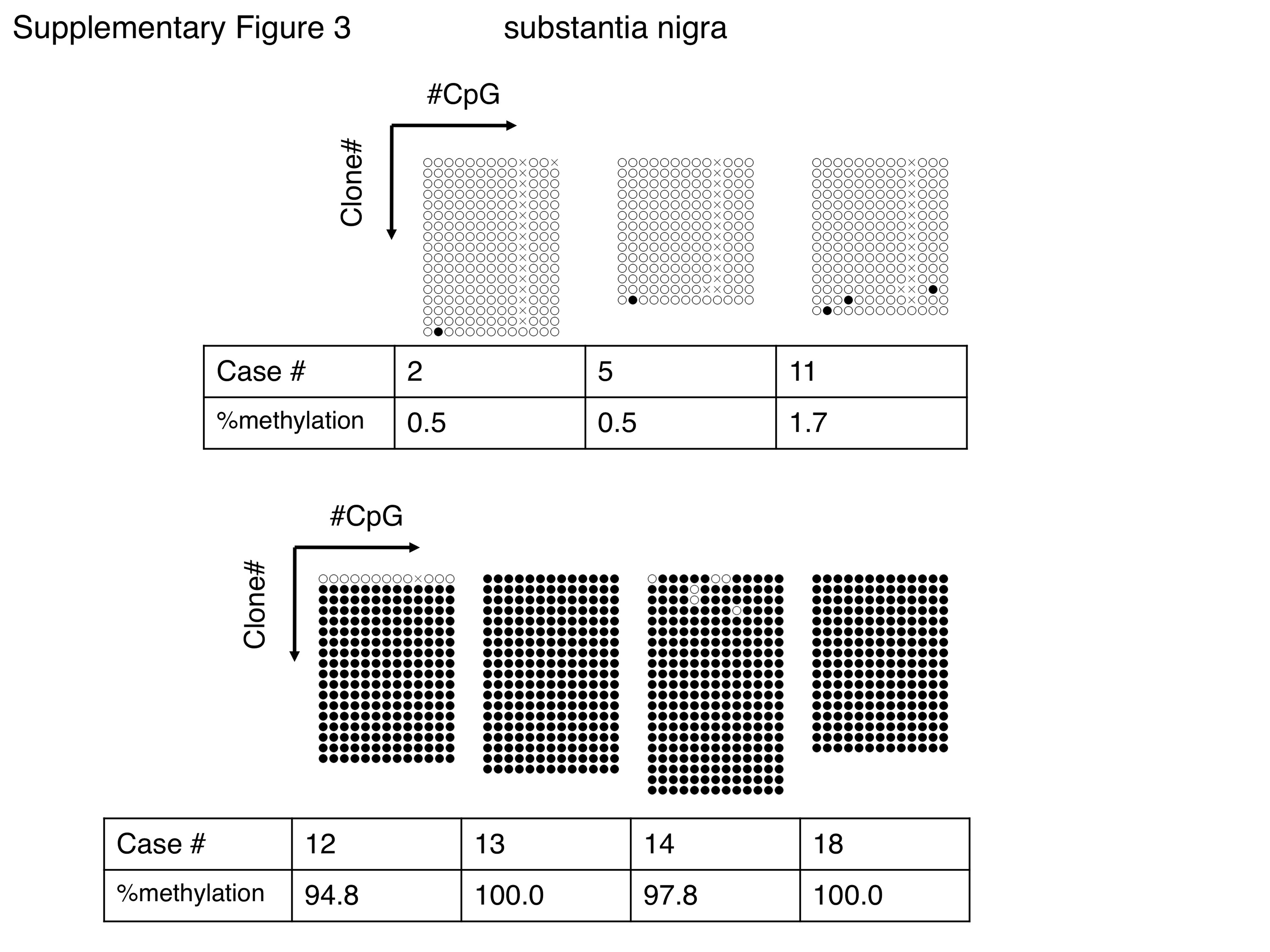

Supplement: Figure S3 — Methylation patterns from substantia nigra samples. (TIF) [file pone.0015522.s003.tif]

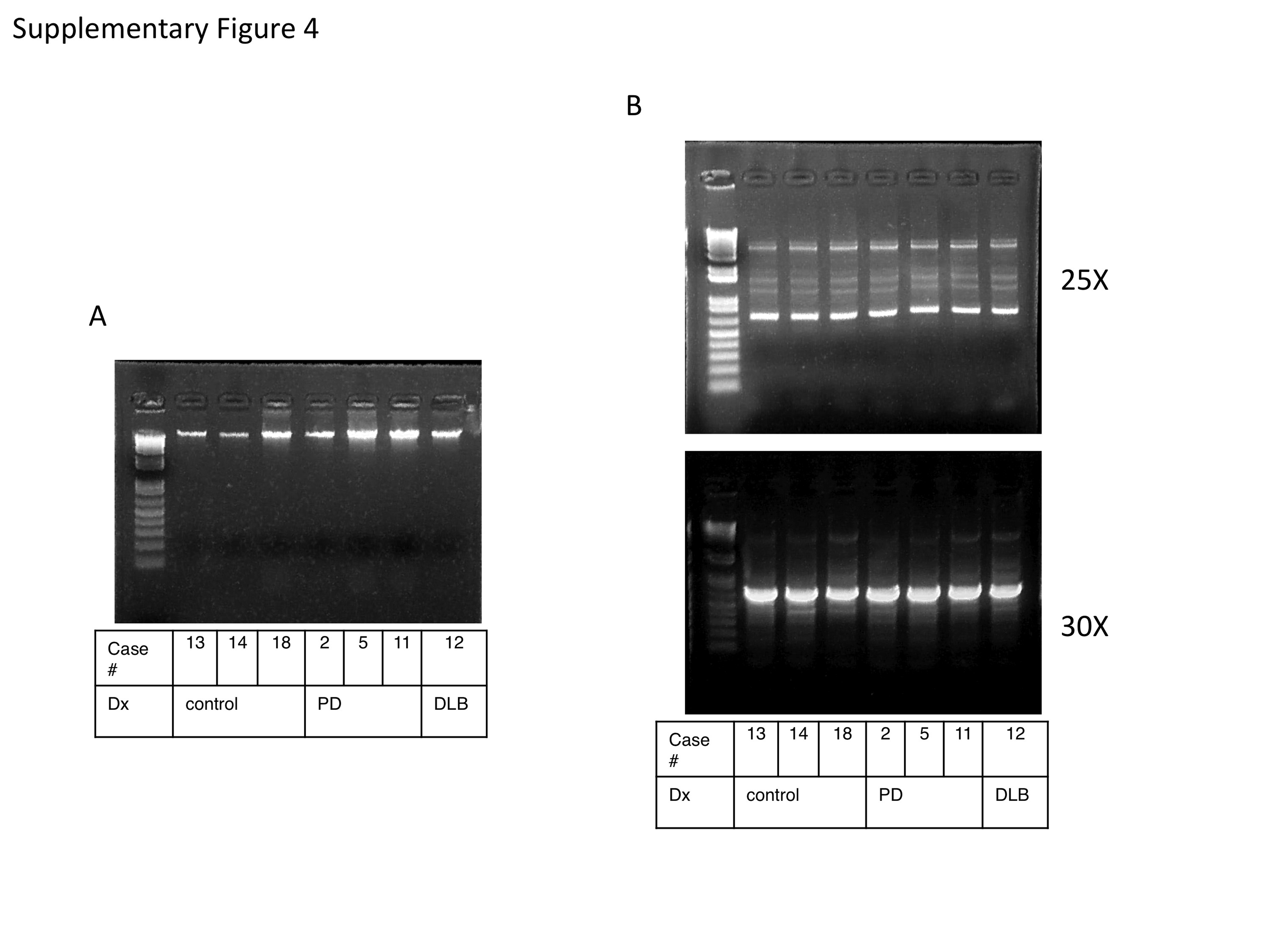

Supplement: Figure S4 — Quality check of genomic DNA from substantia nigra. A: Electrophoresis of 500ng genomic DNA from different substantia nigra. No degradation was observed. B: PCR amplification of CpG-2 region from genomic DNA extracted from substantia nigras. Equal amplification of the region was seen with different PCR cycles. Primers used to clone CpG-2 fragment for luciferase assay experiment was used. (TIF) [file pone.0015522.s004.tif]

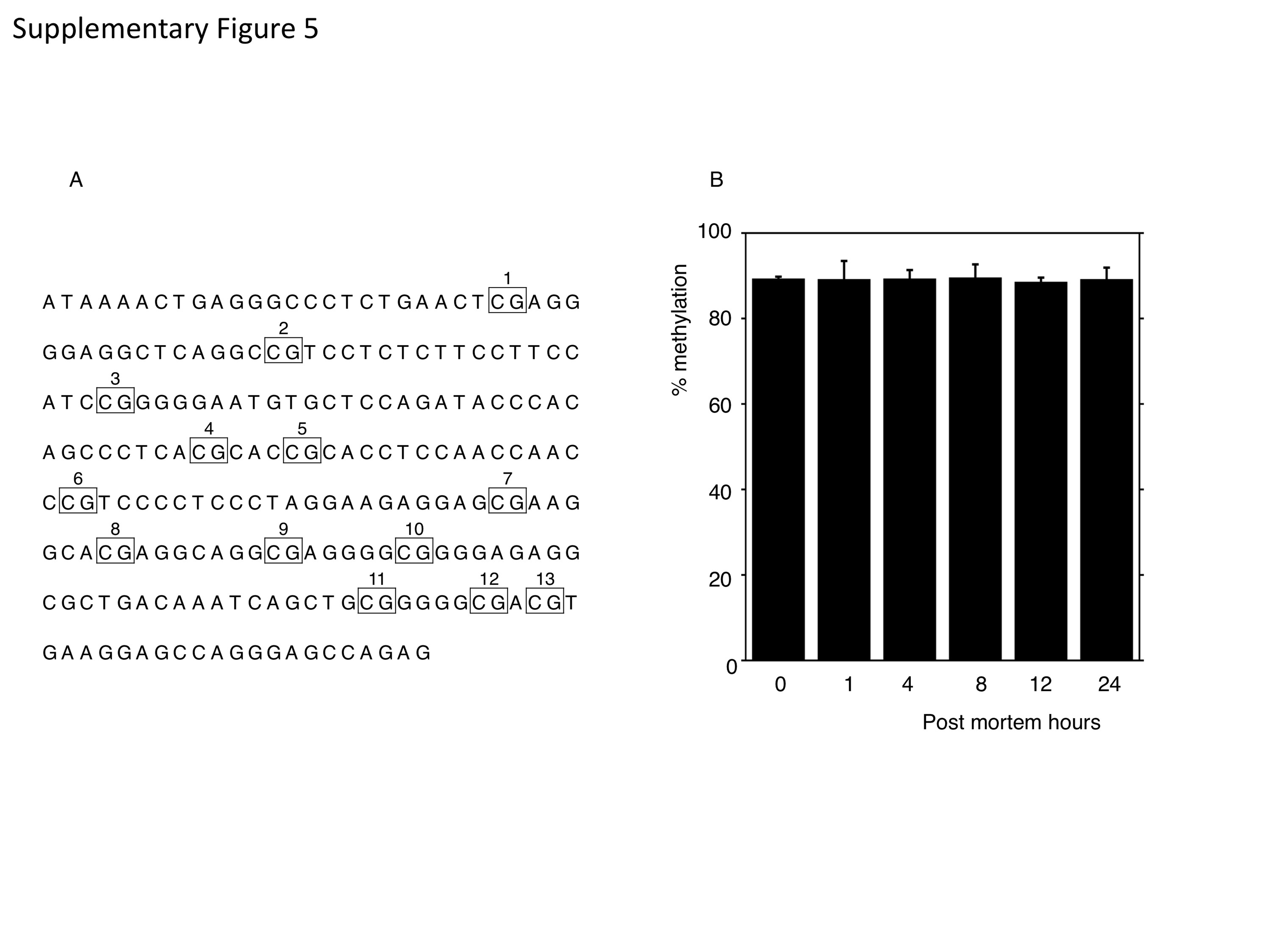

Supplement: Figure S5 — CpG methylation is stable up to 24hours of postmortem time. A: A sequence of mouse SNCA CpG island analyzed is shown. There are 13 CpGs. B: C57/BL mice were sacrificed and kept at RT for indicated hours then the SNCA CpG methylation was analyzed by bisulfite sequencing. Primers used to amplify bisufite converted DNA are 5′-ATAAAATTGAGGGTTTTTTGAATT-3′and 5′-CTCTAACTCCCTAACTCCTTCAC -3′. Three mice were used for each time points. Bars are SEs. (TIF) [file pone.0015522.s005.tif]
